# Epidemiological trends and projections of esophageal cancer in BRICS−plus: Based on the GBD 2021 database

**DOI:** 10.3389/fonc.2025.1616702

**Published:** 2025-09-01

**Authors:** Zhenglong Wang, Hongwei Wei, Weifeng Qi, Xiaobo Liu, Hongxue Cui

**Affiliations:** ^1^ School of Clinical Medicine, Shandong Second Medical University, Weifang, China; ^2^ Department of Cardiovascular Surgery, Affiliated Hospital of Shandong Second Medical University, Weifang, China; ^3^ Department of Gynecology, Affiliated Hospital of Qingdao University, Qingdao, China; ^4^ Department of Thoracic Surgery, Affiliated Hospital of Shandong Second Medical University, Weifang, China

**Keywords:** esophageal cancer, BRICS-plus, GBD 2021, health inequalities, projection

## Abstract

**Introduction:**

Esophageal cancer represents a substantial global health challenge. Given their diverse socio-economic profiles and large populations, the BRICS countries are pivotal in elucidating the burden of this disease. Nonetheless, limited research has systematically examined the trends of esophageal cancer within these nations.

**Method:**

This research utilized data from the GBD database, encompassing ASIR, ASPR, ASMR, ASDR, and 95% UI. The EAPC was employed to assess trends, while the BAPC model was used to project future trends. Four risk factors were examined, and health inequalities were evaluated using SII and CI.

**Result:**

In 2021, China reported the highest rates across all metrics among BRICS countries, whereas Egypt exhibited the lowest rates in most cases. Smoking was identified as the predominant factor contributing to esophageal cancer mortality and DALYs in the majority of countries. Ethiopia demonstrated the highest proportion of cases attributable to a diet low in vegetables, India to tobacco chewing, and Russia to alcohol consumption. Health inequalities between countries were observed to be gradually narrowing. Projections indicate that from 2021 to 2030, Egypt, Iran, and South Africa will experience declines across all rates. Brazil, Russia, India, and the UAE are expected to see reductions in ASIR, ASPR, and ASDR but increases in ASMR. Conversely, China’s ASIR, ASPR, and ASMR are projected to rise, except for ASDR. In Saudi Arabia, ASPR and ASMR are anticipated to increase, while ASIR and ASDR will decrease. Ethiopia is expected to witness increases in most rates.

**Conclusion:**

This study examined the burden of esophageal cancer in BRICS countries. Recognizing the disparities across multiple dimensions can aid these nations in formulating more effective public health strategies and optimizing resource allocation, both of which are essential for the prevention and control of the disease.

## Introduction

Esophageal (or esophageal) cancer is a malignant tumor arising in the esophagus, initiated by abnormal proliferation of cells in its lining. Histologically, it is primarily classified into two major types: esophageal squamous cell carcinoma (ESCC, accounting for approximately 84% of cases) and esophageal adenocarcinoma (EAC, comprising roughly 15% of cases) (1), which have analogous and distinct etiologies with modifiable risk factors (tobacco, alcohol use, obesity, diet) (2). Globally, esophageal cancer ranks as the seventh most common cancer and the sixth leading cause of cancer-related deaths (3). In 2013, approximately 442,000 new cases were diagnosed, resulting in 440,000 deaths (4). GLOBOCAN data from 2020 reported 604,000 new cases and 544,000 deaths (3). Given its high incidence and mortality rates, esophageal cancer continues to be a significant global health concern (5).

Notable global regions with high esophageal cancer rates include the Asian Esophageal Cancer Belt (extending from northern Iran and Kazakhstan to parts of China) and the African Esophageal Cancer Corridor (stretching from Ethiopia to South Africa and encompassing some BRICS countries). Focusing on BRICS is essential given their distinctive socio-economic characteristics, large populations, and recent substantial changes (6). Collectively, these countries account for a substantial portion of the global population and are experiencing socio-economic transformations that may influence physical health in ways distinct from those in developed regions (7). Initially, BRICS comprised Brazil, Russia, India, China, and South Africa (8). As of January 1, 2024, five additional countries—Saudi Arabia, Egypt, the UAE, Iran, and Ethiopia—have been added to the group (referred to as BRICS-plus) (9, 10). The economic growth rates in BRICS are relatively high, and the healthcare system reforms they have implemented vary, including measures such as tobacco bans. Investigating esophageal cancer in BRICS facilitates an understanding of how risk factors, healthcare systems, and socio-economic disparities influence physical health within large, culturally diverse populations.

Previous studies have documented the global burden and trends of esophageal cancer. However, limited research has specifically analyzed esophageal cancer trends within BRICS countries (11–13). This study addresses this gap by utilizing GBD 2021 data for an epidemiological analysis of BRICS countries. Through the incorporation of country-specific details, it enhances the precision of epidemiological insights, thereby informing more targeted public health strategies.

## Method

### Data acquisition

Age-standardized rates (ASR) of esophageal cancer incidence (ASIR), prevalence (ASPR), mortality (ASMR), and DALYs (ASDR), along with their corresponding 95% uncertainty intervals (95% UI), for the BRICS countries (Brazil, Russia, India, China, South Africa, Saudi Arabia, Egypt, UAE, Iran, and Ethiopia) during the years 1990–2021 were extracted from the GBD database (14). The latest epidemiological data and refined, standardized methodologies were employed in the data collection process (15). The Institutional Review Board of the University of Washington granted a waiver for informed consent regarding GBD data access (16).

### Statistical analyses

This study employed the Estimated Annual Percentage Change (EAPC) to evaluate trends in ASIR, ASPR, ASMR, and ASDR. EAPCs were derived from a regression model (Y=α+βX+e) applied to ASR over time (17). The EAPC formula is expressed as 100×[exp(β)–1]. A linear regression model was used to compute the 95% confidence interval (CI) for EAPC (18). If both the EAPC and its 95% CI lower limit are positive, ASR exhibits an upward trend; if both the EAPC and its 95% CI upper limit are negative, ASR shows a downward trend; otherwise, ASR remains stable. To project future esophageal cancer burden trends, a Bayesian age-period-cohort (BAPC) model with integrated nested Laplace approximations was employed. Prior research has demonstrated that BAPC provides more accurate estimates compared to alternative methods (19–21).

### Risk factors

GBD 2021 provided a comprehensive assessment of the DALYs and mortality attributable to 87 risk factors (16). According to the GBD database, esophageal cancer as a disease outcome has attributable risks that can be estimated for only four risk factors (22). These include a diet low in vegetables (defined as average daily vegetable consumption), alcohol use (defined as average daily consumption of pure alcohol among current drinkers who consumed alcohol within the past 12 months), smoking (defined as the prevalence of current or former use of any smoked tobacco product), and chewing tobacco (defined as current use of any chewing tobacco product) (22), which were classified as behavioral risks. The analysis integrated data on DALYs and mortality associated with esophageal cancer attributable to these factors.

### Health inequalities analysis

To assess health inequalities in BRICS countries, the Slope Index of Inequality (SII) and Concentration Index (CI) were employed (23). The SII represents the slope of the regression line connecting four esophageal-cancer-related indicators to the weighted rank of BRICS countries (24). The CI, a value ranging from -1 to 1 calculated by integrating the area under the curve, evaluates relative differences in esophageal cancer burden across countries (24). A negative CI indicates a higher esophageal cancer burden in countries with lower Socio-demographic Index (SDI) values. The SDI ranges from 0 to 1, with higher values reflecting greater socioeconomic development (15).

## Result

**Figure 1:**
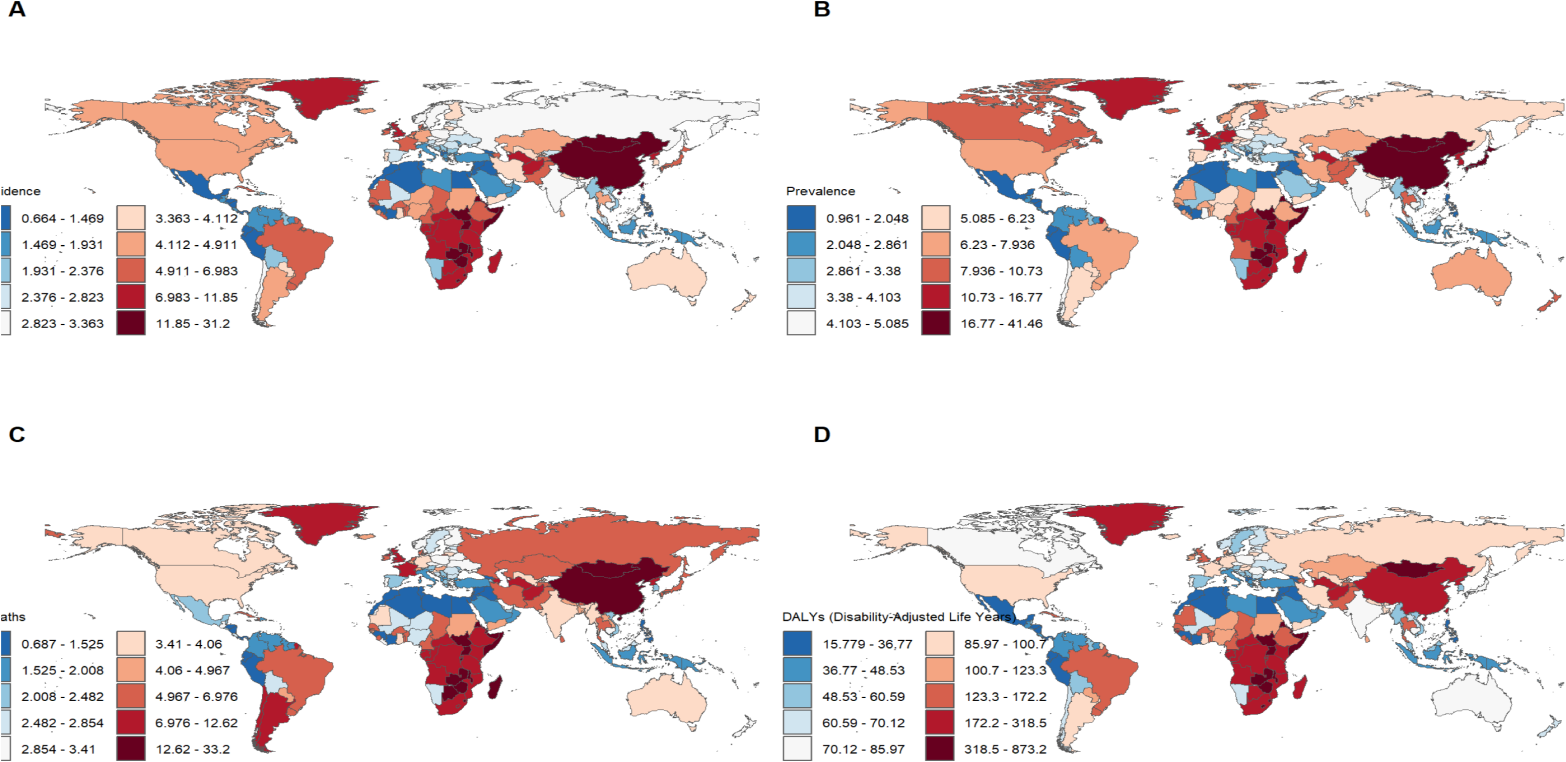
Burden of disease for esophageal cancer in 204 countries and territories in 2021. **(A)** ASIR; **(B)** ASPR; **(C)** ASMR; **(D)** ASDR.

**Figure 2:**
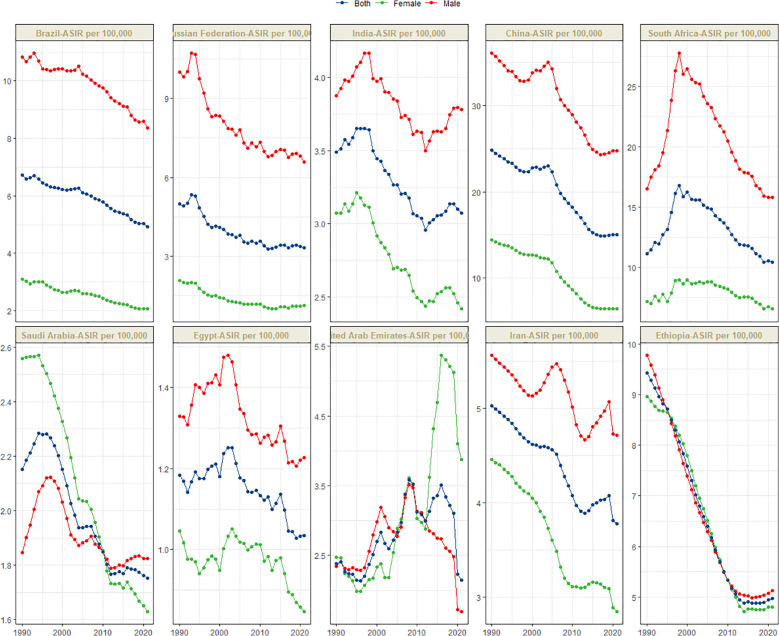
Time trend of ASIR in esophageal cancer in BRICS from 1990 to 2021.

**Figure 3:**
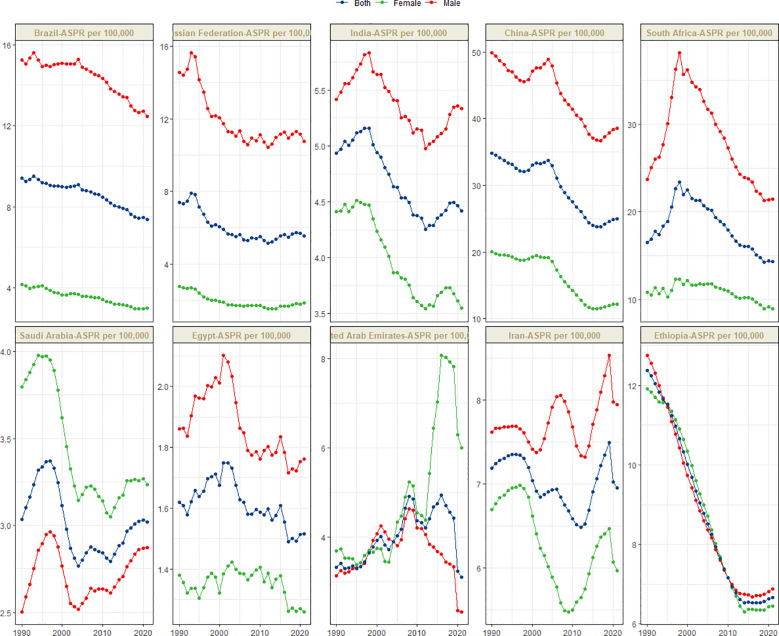
Time trend of ASPR in esophageal cancer in BRICS from 1990 to 2021.

**Figure 4:**
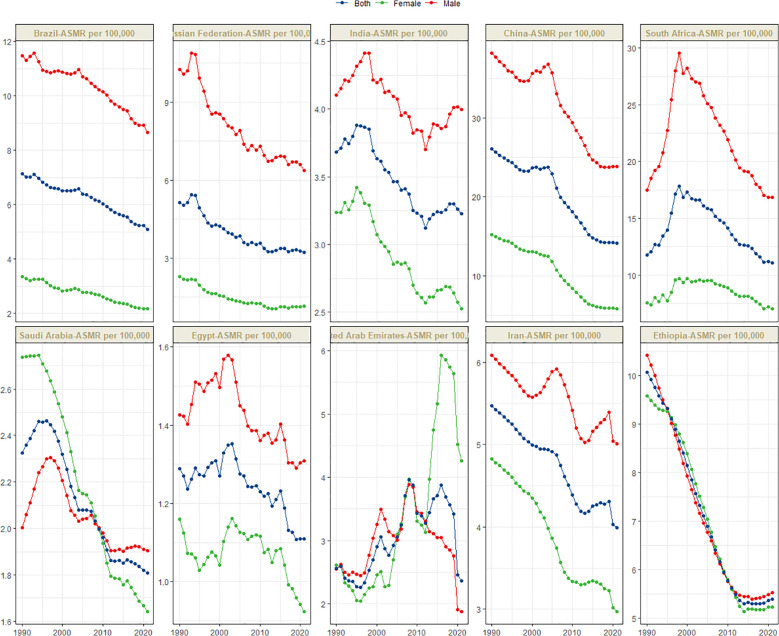
Time trend of ASMR in esophageal cancer in BRICS from 1990 to 2021.

**Figure 5:**
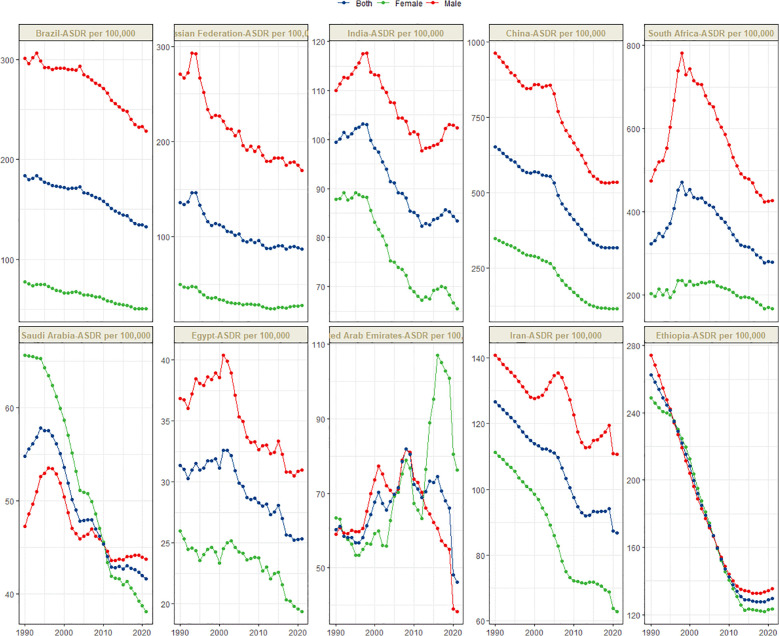
Time trend of ASDR in esophageal cancer in BRICS from 1990 to 2021.

**Figure 1** presents the global distribution of ASIR, ASPR, ASMR, and ASDR for esophageal cancer. **Figures 2**–**5** depict the temporal trends of ASIR, ASPR, ASMR, and ASDR of esophageal cancer from 1990 to 2021. By comparing data from 1990 and 2021, BRICS countries generally exhibit a downward trend in ASIR, ASPR, ASMR, and ASDR. Among these, Brazil, the Russian Federation, China, and Ethiopia demonstrate a gradual decline over the years. In contrast, other BRICS countries experienced fluctuating declines.

In 2021, China had the highest esophageal cancer incidence among BRICS countries, with an ASIR of 15.04 per 100,000 (95% UI: 12.04–18.43), ranking 9th globally. Egypt exhibited the lowest ASIR at 1.03 per 100,000 (95% UI: 0.82–1.30) (**Figure 1A**). Compared to 1990, the UAE showed an increasing trend in esophageal cancer ASIR (EAPC = 1.11, 95% CI: 0.58–1.64), whereas Ethiopia experienced the sharpest decline (EAPC = -2.5, 95% CI: -2.69 to -2.31) (**Figure 2**, **Table 1**). In 2021, excluding China and South Africa, the esophageal cancer ASIR in other BRICS countries was below the global average of 6.65 per 100,000 (95% UI: 5.88–7.45).

**Table 1:** ASIR and trends of esophageal cancer in BRICS.

Country	Age-standardized incidence per 100,000 population (95% UI)		EAPC (95% CI)	AAPC (95% CI)
	1990	2021		
Brazil	6.71 (6.39, 6.93)	4.93 (4.65, 5.14)	-0.97 (-1.05, -0.89)	-1.01 (-1.16 - -0.87)
Russian Federation	4.99 (4.88, 5.08)	3.33 (3.01, 3.61)	-1.51 (-1.74 to -1.28)	-1.15 (-1.73 - -0.56)
India	3.49 (3.02, 4.38)	3.07 (2.69, 3.69)	-0.66 (-0.78 to -0.54)	-0.41 (-0.61 - -0.21)
China	24.80 (20.71, 20.71)	15.04 (12.04, 18.43)	-1.88 (-2.09 to -1.67)	-1.60 (-1.78 - -1.42)
South Africa	11.16 (9.82, 13.42)	10.43 (9.45, 11.60)	-0.73 (-1.23 to -0.23)	-0.34 (-1.05 - 0.38)
Saudi Arabia	2.15 (1.56, 2.87)	1.75 (1.38, 2.23)	-0.99 (-1.11 to -0.87)	-0.66 (-0.80 - -0.52)
Egypt	1.18 (1.02, 1.43)	1.03 (0.82, 1.30)	-0.45 (-0.58 to -0.32)	-0.36 (-0.87 - 0.15)
United Arab Emirates	2.38 (1.75, 3.19)	2.14 (1.65, 2.67)	1.11 (0.58 to 1.64)	-0.49 (-2.52 - 1.59)
Iran	5.02 (4.28, 5.64)	3.78 (3.40, 4.11)	-0.93 (-1.02 to -0.84)	-0.93 (-1.07 - -0.79)
Ethiopia	9.42 (6.65, 12.21)	4.97 (4.01, 6.66)	-2.5 (-2.69 to -2.31)	-2.04 (-2.17 - -1.91)
Global	8.86 (7.96, 9.69)	6.65 (5.88, 7.45)	-1.12 (-1.25 to -1)	-0.94 (-1.06 - -0.82)

In 2021, China had the highest esophageal cancer prevalence among BRICS countries, with an ASPR of 24.99 per 100,000 (95% UI: 19.95–30.69), ranking second globally. Egypt exhibited the lowest ASPR at 1.51 per 100,000 (95% UI: 1.19–1.92) (**Figure 1B**). Compared to 1990, the UAE showed an increasing trend in esophageal cancer ASPR (EAPC = 0.92, 95% CI: 0.47–1.38), whereas Ethiopia experienced the sharpest decline (EAPC = -2.44, 95% CI: -2.62 to -2.25) (**Figure 3**, **Table 2**). In 2021, excluding China and South Africa, the esophageal cancer ASPR in other BRICS countries was below the global average of 11.47 per 100,000 (95% UI: 10.15–12.80).

**Table 2:** ASPR and trends of esophageal cancer in BRICS.

Country	Age-standardized prevalence per 100,000 population (95% UI)		EAPC (95% CI)	AAPC (95% CI)
	1990	2021		
Brazil	9.42 (9.05, 9.69)	7.37 (7.00, 7.68)	-0.8 (-0.89, -0.71)	-0.82 (-1.04 - -0.60)
Russian Federation	7.38 (7.24, 7.51)	5.55 (5.01, 6.04)	-1.07 (-1.36 to -0.78)	-0.84 (-1.53 - -0.14)
India	4.93 (4.30, 6.15)	4.42 (3.88, 5.26)	-0.61 (-0.73 to -0.49)	-0.36 (-0.52 - -0.20)
China	34.86 (28.91, 40.57)	24.99 (19.95, 30.69)	-1.36 (-1.56 to -1.16)	-1.07 (-1.23 - -0.92)
South Africa	16.46 (14.57, 19.45)	14.34 (13.06, 16.07)	-0.97 (-1.41 to -0.53)	-0.51 (-1.00 - -0.02)
Saudi Arabia	3.04 (2.15, 4.10)	3.02 (2.35, 3.90)	-0.37 (-0.57 to -0.17)	-0.01 (-0.14 - 0.12)
Egypt	1.62 (1.41, 1.90)	1.51 (1.19, 1.92)	-0.32 (-0.45 to -0.2)	-0.15 (-0.59 - 0.30)
United Arab Emirates	3.33 (2.42, 4.48)	3.11 (2.42, 3.92)	0.92 (0.47 to 1.38)	-0.24 (-1.98 - 1.53)
Iran	7.18 (6.11, 8.05)	6.95 (6.25, 7.64)	-0.18 (-0.32 to -0.03)	-0.16 (-0.27 - -0.05)
Ethiopia	12.38 (8.66, 15.99)	6.67 (5.35, 8.89)	-2.44 (-2.62 to -2.25)	-1.98 (-2.10 - -1.87)
Global	13.34 (11.94, 14.61)	11.47 (10.15, 12.80)	-0.64 (-0.79 to -0.5)	-0.48 (-0.71 - -0.26)

In 2021, China had the highest esophageal cancer mortality among BRICS countries, with an ASMR of 14.13 per 100,000 (95% UI: 11.36–17.18), ranking 12th globally. Egypt exhibited the lowest ASMR at 1.11 per 100,000 (95% UI: 0.88–1.39) (**Figure 1C**). Compared to 1990, the UAE showed an increasing trend in esophageal cancer ASMR (EAPC = 1.27, 95% CI: 0.72–1.82), whereas Ethiopia experienced the sharpest decline (EAPC = -2.44, 95% CI: -2.63 to -2.26) (**Figure 4**, **Table 3**). In 2021, excluding China and South Africa, the esophageal cancer ASMR in other BRICS countries was below the global average of 6.25 per 100,000 (95% UI: 5.53–7.00).

**Table 3:** ASMR and trends in esophageal cancer in BRICS.

Country	Age-standardized mortality per 100,000 population (95% UI)		EAPC (95% CI)	AAPC (95% CI)
	1990	2021		
Brazil	7.14 (6.77, 7.37)	5.09 (4.80, 5.32)	-1.05 (-1.12, -0.97)	-1.09 (-1.24 - -0.95)
Russian Federation	5.14 (5.02, 5.23)	3.22 (2.93, 3.49)	-1.72 (-1.93 to -1.52)	-1.37 (-1.95 - -0.78)
India	3.68 (3.18, 4.65)	3.23 (2.83, 3.89)	-0.67 (-0.79 to -0.56)	-0.43 (-0.65 - -0.21)
China	26.06 (21.77, 30.10)	14.13 (11.36, 17.18)	-2.26 (-2.5 to -2.02)	-0.43 (-0.51 - -0.35)
South Africa	11.75 (10.35, 14.28)	11.06 (10.06, 12.30)	-0.7 (-1.21 to -0.19)	-0.29 (-0.96 - 0.39)
Saudi Arabia	2.32 (1.70, 3.07)	1.81 (1.43, 2.33)	-1.15 (-1.27 to -1.02)	-0.80 (-0.95 - -0.66)
Egypt	1.29 (1.11, 1.57)	1.11 (0.88, 1.39)	-0.46 (-0.59 to -0.32)	-0.57 (-0.98 - -0.16)
United Arab Emirates	2.56 (1.90, 3.40)	2.36 (1.85, 2.93)	1.27 (0.72 to 1.82)	-0.41 (-2.50 - 1.73)
Iran	5.47 (4.65, 6.14)	3.99 (3.58, 4.35)	-1.01 (-1.1 to -0.92)	-1.06 (-1.15 - -0.97)
Ethiopia	10.06 (7.10, 13.03)	5.38 (4.35, 7.22)	-2.44 (-2.63 to -2.26)	-1.99 (-2.11 - -1.87)
Global	9.02 (8.11, 9.87)	6.25 (5.53, 7.00)	-1.41 (-1.55 to -1.27)	-1.19 (-1.36 - -1.01)

In 2021, China had the highest esophageal cancer DALYs among BRICS countries, with an ASDR of 317.184 per 100,000 (95% UI: 252.46–392.42), ranking 14th globally. Egypt exhibited the lowest ASDR at 25.33 per 100,000 (95% UI: 19.97–33.93) (**Figure 1D**). Compared to 1990, the UAE showed a slight increasing trend in esophageal cancer ASDR (EAPC = 0.32, 95% CI: -0.17 to 0.81), whereas Ethiopia experienced the sharpest decline (EAPC = -2.74, 95% CI: -2.94 to -2.55) (**Figure 5**, **Table 4**). In 2021, excluding China and South Africa, the esophageal cancer ASDR in other BRICS countries was below the global average of 148.56 per 100,000 (95% UI: 131.71–166.82).

**Table 4:** ASDR and trends in esophageal cancer in BRICS.

Country	Age-standardized DALYs per 100,000 population (95% UI)		EAPC (95% CI)	AAPC (95% CI)
	1990	2021		
Brazil	183.52 (176.81, 188.74)	132.78 (126.26, 138.35)	-1.06 (-1.15 to -0.96)	-1.08 (-1.25 - -0.91)
Russian Federation	136.17 (133.43, 138.67)	86.83 (78.57, 94.59)	-1.68 (-1.9 to -1.47)	-1.31 (-1.90 - -0.71)
India	99.41 (86.45, 124.39)	83.40 (73.11, 99.11)	-0.82 (-0.94 to -0.7)	-0.57 (-0.73 - -0.41)
China	653.31 (543.18, 758.88)	317.18 (252.46, 392.42)	-2.67 (-2.9 to -2.44)	-2.31 (-2.46 - -2.16)
South Africa	322.87 (286.32, 380.88)	279.79 (254.98, 313.69)	-1 (-1.48 to -0.5)	-0.53 (-0.99 - -0.07)
Saudi Arabia	54.80 (39.31, 73.76)	41.63 (32.40, 54.55)	-1.21 (-1.33 to -1.09)	-0.88 (-1.03 - -0.72)
Egypt	31.36 (27.39, 36.69)	25.33 (19.97, 31.93)	-0.77 (-0.91 to -0.63)	-0.63 (-1.13 - -0.13)
United Arab Emirates	60.25 (44.32, 81.00)	46.26 (35.71, 58.38)	0.32 (-0.17 to 0.81)	-0.98 (-2.88 - 0.95)
Iran	126.69 (108.34, 141.56)	86.71 (78.90, 94.51)	-1.24 (-1.33 to -1.15)	-1.23 (-1.36 - -1.10)
Ethiopia	262.78 (183.93, 339.39)	129.60 (104.33, 172.95)	-2.74 (-2.94 to -2.55)	-2.25 (-2.37 - -2.14)
Global	235.32 (210.52, 258.68)	148.56 (131.71, 166.82)	-1.73 (-1.87 to -1.59)	-1.47 (-1.61 - -1.33)

In most BRICS countries, the ASIR, ASPR, ASMR, and ASDR of esophageal cancer were higher in males. However, in Saudi Arabia before 2010, the female ASIR, ASMR, and ASDR were greater, while the female ASPR was higher during 1990–2021. In the UAE, after 2013, the female ASIR, ASPR, ASMR, and ASDR were significantly higher than those in males. In Ethiopia, from 1990 to 2021, the ASIR, ASPR, ASMR, and ASDR were similar between females and males.

### Health inequality

**Figure 6:**
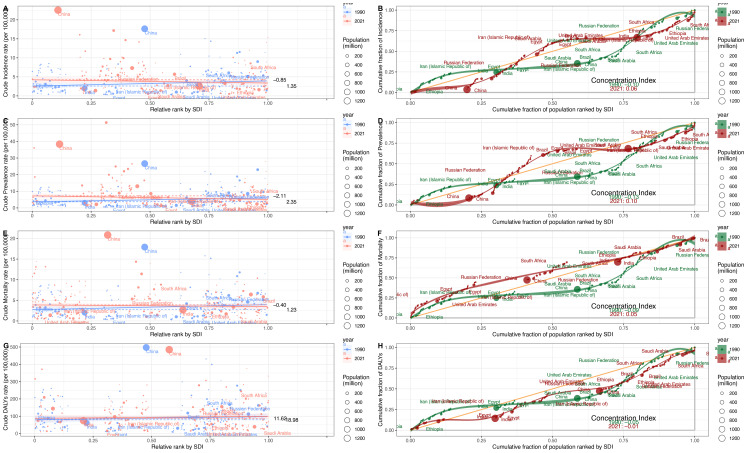
Health inequality regression curves and Concentration curves of esophageal cancer, 1990–2021. **(A, B)** ASIR. **(C, D)** ASPR. **(E, F)** ASMR. **(G, H)** ASDR.

In 1990 and 2021, the SII values for ASIR were 1.35 in 1990 and -0.85 in 2021 (**Figure 6A**). From 1990 to 2021, the CI for incidence showed an upward trend, starting at -0.1 in 1990 and reaching 0.06 in 2021 (**Figure 6B**). This change indicates a reduction in the inequality of the ASIR burden of esophageal cancer between high-income and low-income countries during this period.

For the ASPR, the SII values were 2.35 in 1990 and -2.11 in 2021 (**Figure 6C**). Between 1990 and 2021, the CI for prevalence exhibited an upward trend, with values of -0.13 in 1990 and 0.10 in 2021 (**Figure 6D**). This trend suggests a shift toward reduced inequality in the ASPR burden among different countries during this period.

For the ASMR, the SII values were 1.23 in 1990 and -0.4 in 2021 (**Figure 6E**). From 1990 to 2021, the CI for mortality exhibited an upward trend, with values of -0.09 in 1990 and 0.05 in 2021 (**Figure 6F**). This indicates a reduction in the inequality of the ASMR burden among different countries during this period.

For the ASDR, the SII values were 18.98 in 1990 and 11.62 in 2021, indicating a positive correlation between ASDR and the SDI (**Figure 6G**). Between 1990 and 2021, the CI for ASDR exhibited an upward trend, with values of -0.09 in 1990 and 0.05 in 2021 (**Figure 6H**). This suggests a reduction in the inequality of the ASDR burden among different countries during this period.

Despite a regional reduction in the inequality of the esophageal cancer burden between low-income and high-income countries, inequality persists. Although the economic gap has narrowed in some regions, global disparities in esophageal cancer remain a continuing concern.

### Risk factors

In 2021, within the category of level 3 risk factors, for all BRICS countries except Ethiopia, South Africa, and India, the proportion of esophageal cancer mortality attributable to smoking, expressed as a percentage of total esophageal cancer mortality, was the highest. Similarly, for these same BRICS countries (excluding Ethiopia, South Africa, and India), the proportion of DALYs attributed to smoking risk, as a percentage of total DALYs, was also the highest.

**Figure 7:**
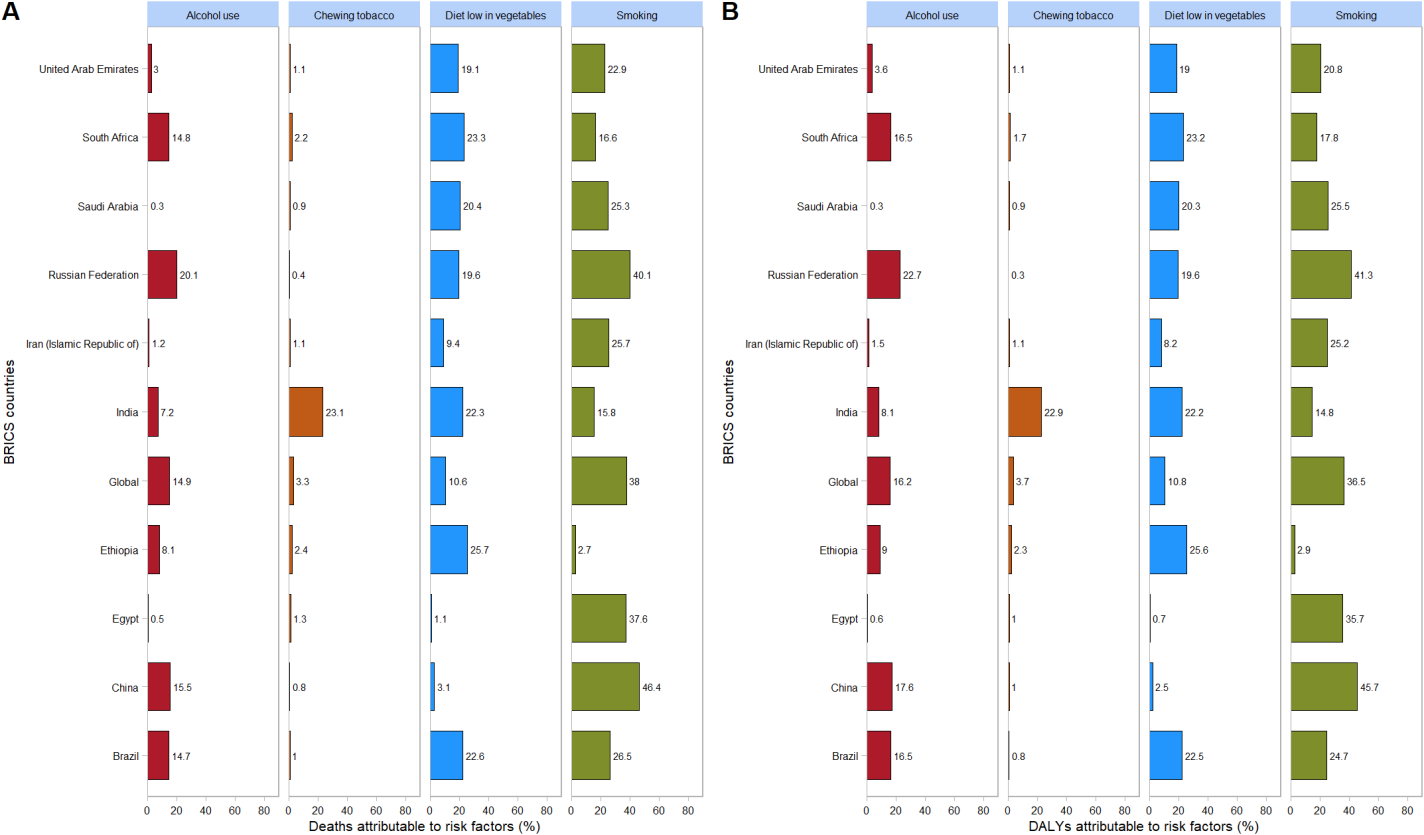
Proportion of esophageal cancer DALYs attributable to tobacco chewing, smoking, alcohol use, and diet low in vegetables, for BRICS countries, 2021. **(A)** Mortality; **(B)** DALYs.

In 2021, among BRICS countries, China had the highest proportion of esophageal cancer mortality attributable to smoking (46.4%). Ethiopia had the highest proportion attributable to a diet low in vegetables (25.7%). India had the highest proportion associated with chewing tobacco (23.1%). The Russian Federation had the highest proportion attributable to alcohol use (20.1%) (**Figure 7A**).

In 2021, among BRICS countries, China had the highest proportion of esophageal cancer DALYs attributable to smoking (45.7%). Ethiopia had the highest proportion attributable to a diet low in vegetables (25.6%). India had the highest proportion associated with chewing tobacco (22.9%). The Russian Federation had the highest proportion attributable to alcohol use (22.7%) (**Figure 7B**).

### Projection of disease burden trends

Based on the BAPC projection (see **Supplementary Material**, **Supplementary Figures S1–S10**), from 2021 to 2030, Egypt, Iran, and South Africa are expected to exhibit a declining trend in ASIR, ASPR, ASMR, and ASDR. Meanwhile, during the same period, Brazil, Russia, India, and the UAE will experience a decreasing trend in ASIR, ASPR, and ASDR but an increasing trend in ASMR.

**Figure 8:**
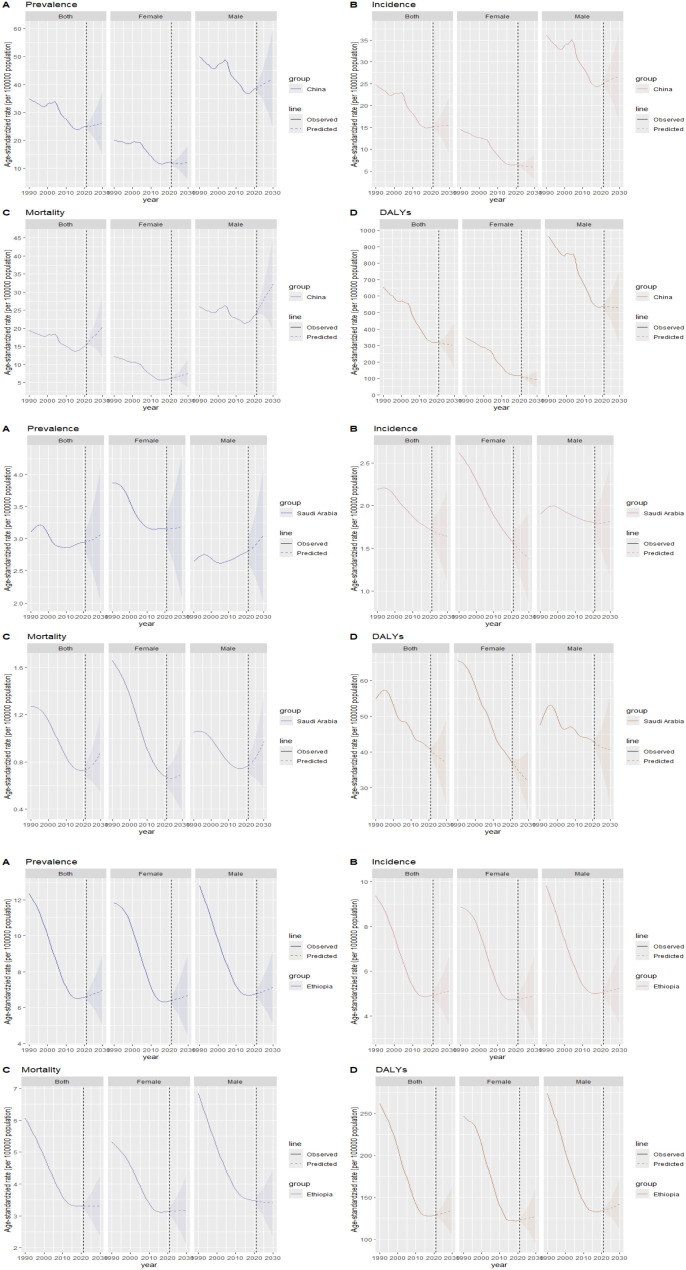
Projection of esophageal cancer ASIR, ASPR, ASMR, and ASDR trends in China, Saudi Arabia, and Ethiopia from 2021 to 2030. **(A)** ASPR; **(B)** ASIR; **(C)** ASMR; **(D)** ASDR.

By 2030, among BRICS countries, China is projected to have the highest rates of esophageal cancer in terms of ASIR at 15.45/100,000 (95% UI: 8.95, 21.95), ASPR at 26.17/100,000 (95% UI: 14.60, 37.74), ASMR at 20.39/100,000 (95% UI: 11.55, 29.23), and ASDR at 299.34/100,000 (95% UI: 158.30, 440.38). From 2021 to 2030, China’s ASIR, ASPR, and ASMR for esophageal cancer are expected to exhibit an upward trend, whereas its ASDR will show a downward trend during the same period (**Figure 8**).

In Saudi Arabia, by 2030, the ASPR for esophageal cancer is estimated at 3.07/100,000 (95% UI: 1.99, 4.15), and the ASMR is predicted to increase from 2021 to 2030, reaching 0.88/100,000 (95% UI: 0.54, 1.23). Conversely, the ASIR, projected at 1.64/100,000 (95% UI: 1.10, 2.18), and the ASDR, at 37.03/100,000 (95% UI: 25.06, 49.01), are expected to decline during the same period (**Figure 8**).

Regarding Ethiopia, by 2030, the ASIR for esophageal cancer is projected at 5.12/100,000 (95% UI: 3.55, 6.69), the ASPR at 6.97/100,000 (95% UI: 4.97, 8.96), and the ASDR at 134.66/100,000 (95% UI: 104.58, 164.74). These indicators are expected to exhibit an upward trend from 2021 to 2030. In contrast, the ASMR, estimated at 3.31/100,000 (95% UI: 2.33, 4.29), will remain stable during the same period (**Figure 8**).

## Discussion

The BRICS is an international cooperation framework for emerging economies and developing countries, aimed at enhancing economic, political, and social cooperation among member states and reforming the global governance system (9). Currently, the BRICS countries face a multitude of challenges. These include an aging population, inequitable distribution of healthcare resources, and a persistently high smoking rate. Additionally, the rapid pace of economic and social development has contributed to the deterioration of dietary patterns, with unhealthy eating habits becoming increasingly prevalent. This complex situation is likely to increase the incidence, prevalence, and mortality rates of esophageal cancer, as well as the associated DALYs rate.

Although China exhibited negative AAPC and EAPC values for the ASIR, ASPR, and ASMR of esophageal cancer from 1990 to 2021, it still ranked first among BRIC countries in these rates in 2021. Smoking, dietary patterns, and alcohol consumption remain the primary risk factors contributing to the esophageal cancer burden in China (25). Notably, the smoking prevalence among Chinese men is as high as 60%, whereas the proportion of smokers willing to quit is only 16.1% (26). Moreover, the smoking rates among Chinese women and adolescents are increasing (27). Projections indicate that the ASDR of esophageal cancer in China may decline from 2021 to 2030, whereas the ASIR, ASPR, and ASMR are expected to increase during the same period. The gender disparity in the burden of esophageal cancer in China is pronounced. Specifically, the ASIR, ASPR, ASMR, and ASDR for esophageal cancer in men are more than double those in women, plausibly attributable to the significantly higher smoking rate among men (28). In certain regions of China, such as Linxian, the consumption of pickled foods, which are well-established carcinogens (29), is associated with a high incidence of esophageal cancer (30). China faces a significant level of population aging, both globally and within the BRICS context. Consequently, the cancer burden is increasing (31, 32). The uneven distribution of medical resources further exacerbates the burden of esophageal cancer across different regions, populations, and age groups, posing a critical challenge for disease prevention and control efforts in China.

Between 1990 and 2021, the AAPC and EAPC of the ASIR, ASPR, ASMR, and ASDR for esophageal cancer in Brazil were all less than 0. Consequently, Brazil does not fall into the category of high-risk countries for esophageal cancer (33, 34). Despite its advanced socioeconomic status and commendable healthcare indicators, the southern region of Brazil has, over a long period, consistently recorded the highest incidence and mortality rates for esophageal cancer (35). Local practices, such as the consumption of hot mate tea, are widely regarded as significant contributors to this phenomenon (36, 37). According to the BAPC projection, from 2021 to 2030, the ASIR, ASPR, and ASDR are expected to decline, while the ASMR is projected to increase. This anticipated trend may be closely associated with the enforcement of nationwide smoking-ban policies. The successful implementation of anti-tobacco campaigns has effectively reduced both smoking and tobacco chewing behaviors, which are widely acknowledged as major risk factors for lung and esophageal cancers (38, 39). Additionally, comprehensive prevention and control measures, integrating education, treatment, and policy-based strategies, have significantly contributed to the overall reduction in tobacco consumption (40).

Russia, the world’s largest country spanning two continents, witnessed negative AAPC and EAPC in ASIR, ASPR, ASMR, and ASDR for esophageal cancer between 1990 and 2021. The ratification of the Framework Convention on Tobacco Control (FCTC) in 2008 played a significant role in reducing male smoking prevalence and associated mortality. However, female smoking rates in Russia have been increasing steadily, surpassing those of the United Kingdom and the United States in recent years (41). Based on the BAPC projection, from 2021 to 2030, Russia’s ASIR, ASPR, and ASDR are projected to decline, whereas ASMR is expected to remain stable or potentially increase. Among the BRICS countries, Russia exhibits the highest risk levels associated with alcohol use. Despite the reduction in alcohol consumption over the past decades, recent policy efforts to further control it have shown limited progress, raising concerns about potential increases in alcohol consumption in the future (42). Given Russia’s status as a high-alcohol-consuming country, its government should implement measures to increase public awareness of the link between alcohol consumption and cancer risk.

Although India as a whole does not qualify as a high-risk nation for esophageal cancer, specific regions, such as the Kashmir Valley, exhibit remarkably elevated incidence rates (43). The AAPC and EAPC of ASIR, ASPR, ASMR, and ASDR for esophageal cancer in India were all below 0 during the period of 1990-2021. It is projected that ASIR, ASPR, and ASDR will continue to decline from 2021 to 2030, whereas ASMR is anticipated to exhibit an upward trend. This contrasting scenario may be attributed to the stringent tobacco control measures implemented after the introduction of the National Tobacco Control Plan in 2007-08 (42), thereby leading to a reduction in smoking prevalence. In the context of India, chewing tobacco stands out as the predominant risk factor for esophageal cancer, significantly exceeding the levels observed in other BRICS countries. To tackle this issue holistically, the Indian government’s tobacco-control strategies should extend beyond hookahs and cigarettes and prioritize addressing the use of chewing tobacco (44).

Between 1990 and 2021, the AAPC and EAPC of ASIR, ASPR, ASMR, and ASDR for esophageal cancer in South Africa remained consistently negative. Furthermore, based on BAPC projections, these rates are expected to continue decreasing from 2021 to 2030. A study analyzing gender differences in esophageal cancer incidence and mortality in South Africa provides a clearer explanation for the significantly higher burden of esophageal cancer in men compared to women (45). A particular study has demonstrated that the burden of esophageal cancer is significantly higher among South African men than women, largely due to the greater prevalence of tobacco use and alcohol consumption in this demographic. In response to growing health concerns, South Africa introduced stringent tobacco control policies in 1993 as a proactive strategy to reduce associated health risks (46). Research findings have demonstrated that the dietary pattern in South Africa, marked by a preference for wild foods and a relative scarcity of vegetables, is associated with an increased risk of esophageal cancer (47). Additionally, South Africa faces a pronounced wealth disparity, as indicated by its Gini coefficient of 0.67 (48). To effectively prevent esophageal cancer in the future, it is crucial to tackle both the wealth inequality and the inequitable distribution of medical resources.

Saudi Arabia is the only member within the BRICS group where the burden of esophageal cancer is more pronounced among females. From 1990 to 2021, the EAPC and AAPC values for ASIR, ASPR, ASMR, and ASDR associated with esophageal cancer in Saudi Arabia were predominantly negative. According to BAPC projections, ASIR and ASDR are expected to continue declining. In contrast, ASPR and ASMR are anticipated to increase. This contrasting trend may be attributed to Saudi Arabia’s ratification of the WHO Framework Convention on Tobacco Control in 2015 and the subsequent introduction of significant cigarette taxation policies after 2017 (49). Elevating public awareness of the disease, promoting a healthy lifestyle, and lowering the starting age for esophageal cancer screening can substantially enhance early diagnosis and improve prognosis (50–52). To effectively alleviate the burden of esophageal cancer, it is crucial to establish comprehensive national and regional programs, implement multilevel diagnostic strategies, and consider nutritional and lifestyle factors (53, 54).

In Egypt, from 1990 to 2021, the AAPC and the EAPC of ASIR, ASPR, ASMR, and ASDR related to esophageal cancer were all negative. Projections indicate that from 2021 to 2030, these rates will continue to decline. Our findings reveal that, aside from smoking, the contribution of other risk factors for esophageal cancer in Egypt is below 1%. Notably, the prevalence of smoking among Egyptian males is strikingly high, with 47.5% of individuals aged 15 years and older being smokers (55). Socioeconomic factors and cost significantly impact the accessibility of cancer screening and treatment in Egypt (56, 57). Despite the existence of a government-subsidized healthcare system, the majority of Egyptians continue to shoulder significant healthcare expenses. Data from the Egyptian Family Observatory Survey reveals that public healthcare subsidies disproportionately benefit wealthier groups compared to poorer ones (58). Thus, Egypt must address the inequalities stemming from economic factors by implementing targeted policies and initiatives.

The AAPCs of ASIR, ASPR, ASMR, and ASDR for esophageal cancer in the UAE were all below 0 during 1990-2021, whereas the EAPCs were consistently above 0. Projections derived from the BAPC model indicate that between 2021 and 2030, ASIR, ASPR, and ASDR are likely to continue their downward trends. Conversely, ASMR is expected to exhibit an upward trend. Research findings reveal that women experienced a significant rise in the incidence of esophageal cancer from 2013 to 2016, followed by a subsequent decline. Data from 2017 shows that the UAE had the highest proportion of expatriate young male workers compared to other BRICS countries (9). Notably, smoking, especially the increasing popularity of hookah use among females, has shown a concerning upward trend (59). Similar to many other regions, young people in the UAE are facing a significant issue with obesity, which is largely attributed to the consumption of diets lacking essential nutrients (60). Both obesity and smoking have become prominent public health challenges in the UAE. Therefore, addressing the dietary habits of young migrant workers and decreasing the smoking prevalence are essential elements of future strategies for esophageal cancer prevention.

Although Iran is not classified as a high-risk country for esophageal cancer, Golestan Province, located in northern Iran, demonstrates significantly higher incidence rates of esophageal cancer (61, 62). From 1990 to 2021, the AAPC and EAPC of ASIR, ASPR, ASMR, and ASDR for esophageal cancer in Iran were negative. Projections suggest that from 2021 to 2030, the ASIR, ASPR, and ASDR are likely to continue declining, while the ASMR may remain stable or increase slightly. The reduction in disease burden could be attributed to changes in risk factors, including improvements in lifestyle, decreased tobacco consumption, enhanced dietary habits through increased intake of fresh fruits and vegetables, and the adoption of advanced food preservation techniques (63). High consumption of red meat and processed meat has been identified as a potential risk factor for esophageal cancer (64). Although an Iranian study has shown no significant correlation between esophageal cancer and red meat intake (64), it is still essential to promote a diet abundant in fruits and vegetables, rather than one heavily reliant on meat.

Recent investigations have revealed that Ethiopian patients diagnosed with esophageal cancer rarely engage in smoking or alcohol consumption, which are commonly considered risk factors for the disease (65, 66). The research conducted by Wong and colleagues has demonstrated a strong and statistically significant correlation between the incidence and mortality rates of esophageal cancer and a country’s SDI (67). The economic development of a country influences patient health-seeking behavior and lifestyle choices, as well as access to screening and management options for esophageal cancer, ultimately affecting survival rates (68, 69). In low-income settings like Ethiopia, the prognosis for cancer, particularly esophageal cancer, remains exceptionally grim. This poor outcome can be attributed to several interrelated factors: the insufficient availability of diagnostic equipment, the limited range of treatment options, and the tendency of patients to present for medical care only at advanced stages of the disease (70–72). These circumstances may partially explain Ethiopia’s decline in the ASIR, ASPR, ASMR, and ASDR of esophageal cancer by 2021. However, projections based on the BAPC model suggest that from 2021 to 2030, these rates are likely to rise. This potential increase could be attributed to advancements in medical diagnostic technologies alongside economic growth.

The findings of this study provide scientific evidence to support the precise, differentiated, and coordinated development of healthcare systems in BRICS-plus countries. These findings will assist countries in prioritizing key issues, enhancing the efficiency of esophageal cancer prevention and control under resource constraints, and promoting deeper regional healthcare cooperation. However, there are several limitations that warrant acknowledgment. The availability and quality of raw data constrained the estimation of esophageal cancer data. Although major risk factors were taken into account, emerging factors such as environmental pollutants and genetic predispositions could not be fully explored due to data limitations. Additionally, while the BAPC model is robust, it may not fully account for future changes in healthcare infrastructure, policies, or unforeseen socio-political events, particularly in the context of rapidly changing political and health systems, that could influence esophageal cancer trends over the coming decades. Furthermore, the economic and health data of BRICS countries, including Iran, Egypt, and Russia, are particularly susceptible to external economic and political influences.

## Conclusion

This study provides a comprehensive analysis of the esophageal cancer burden in BRICS countries. Recognizing the disparities in living habits, national policies, economic development levels, and aging populations enables BRICS nations to make more informed decisions for future public health infrastructure and resource allocation. For example, enforcing food hygiene regulations and strengthening tobacco control measures could effectively alleviate the esophageal cancer burden. Preventive strategies should focus on high-risk groups, such as women in Saudi Arabia and the UAE, as well as men in other regions.

## Data Availability

Publicly available datasets were analyzed in this study. This data can be found here: https://vizhub.healthdata.org/gbd-results/.
