# Supplementary material for: Epidemiological trends and projections of esophageal cancer in BRICS−plus: Based on the GBD 2021 database
Source: Front Oncol. 2025 Sep 1;15:1616702. doi: 10.3389/fonc.2025.1616702 (PMC12402698; doi:10.3389/fonc.2025.1616702)
Supplement: Supplementary file 1 [file DataSheet1.docx]

**
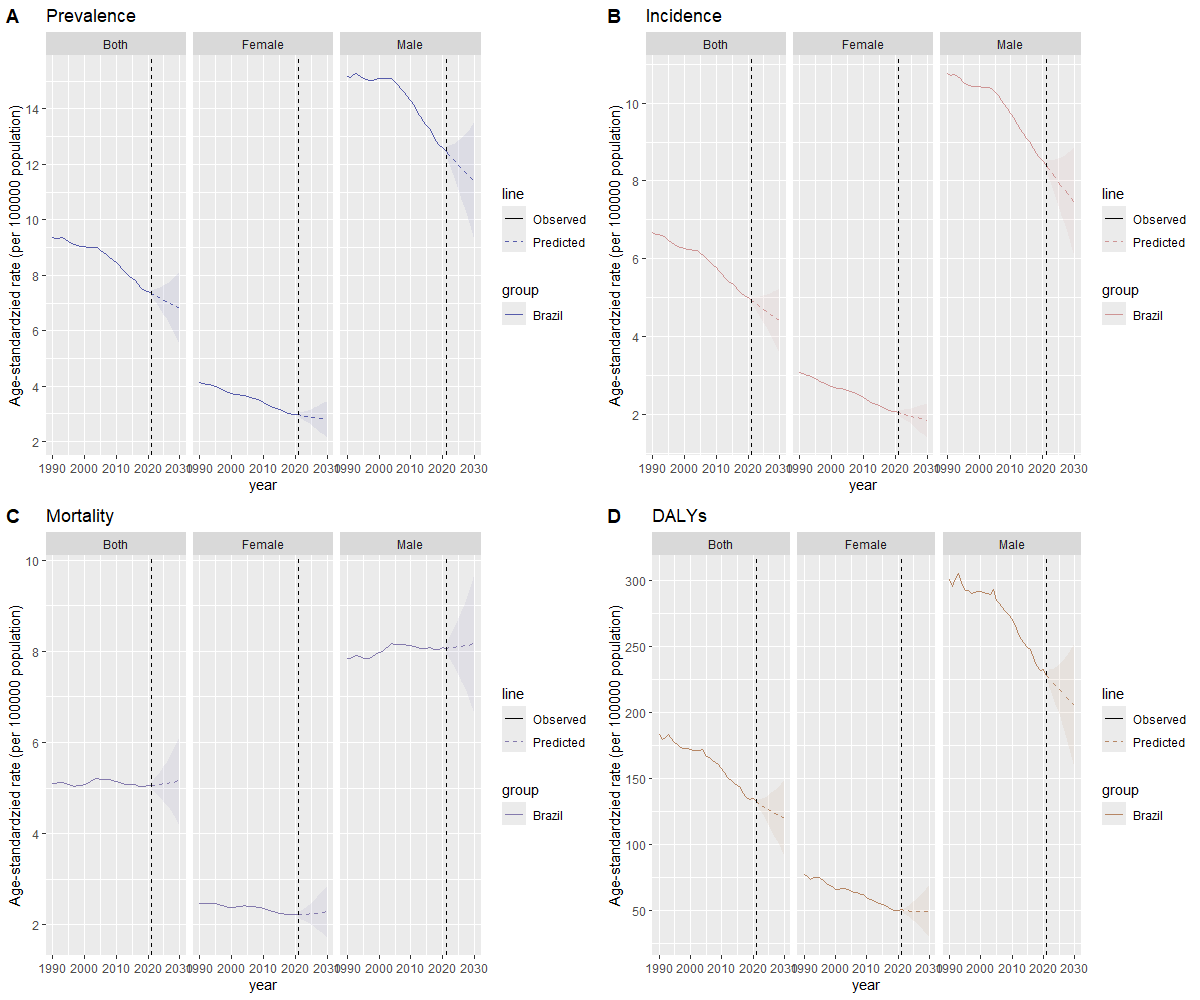
**

**Figure S1 Projection of esophageal cancer ASIR, ASMR, ASMR, and ASDR trends in Brazil from 2021 to 2030.**

**
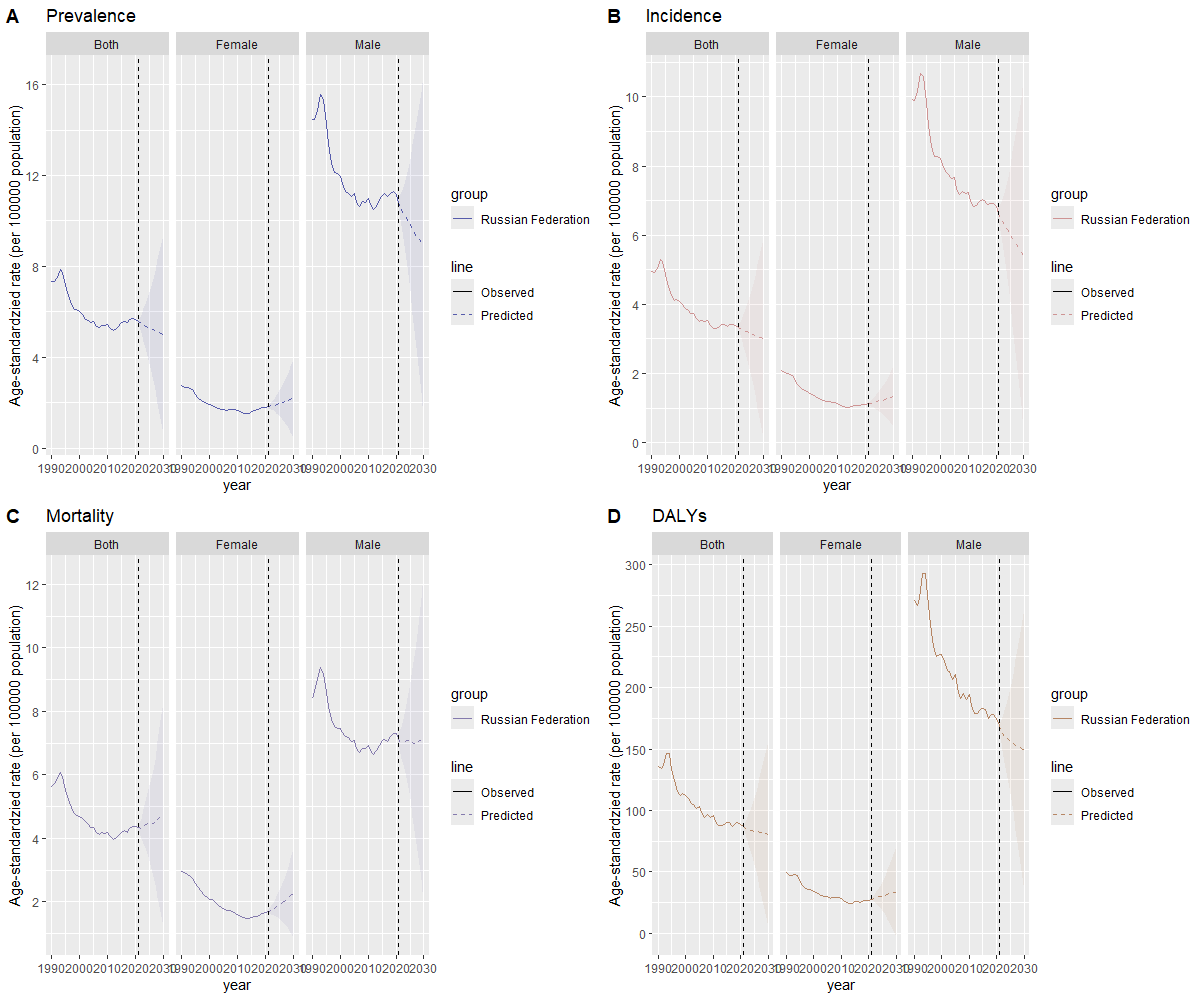
**

**Figure S2 Projection of esophageal cancer ASIR, ASMR, ASMR, and ASDR trends in Russian Federation from 2021 to 2030.**

**
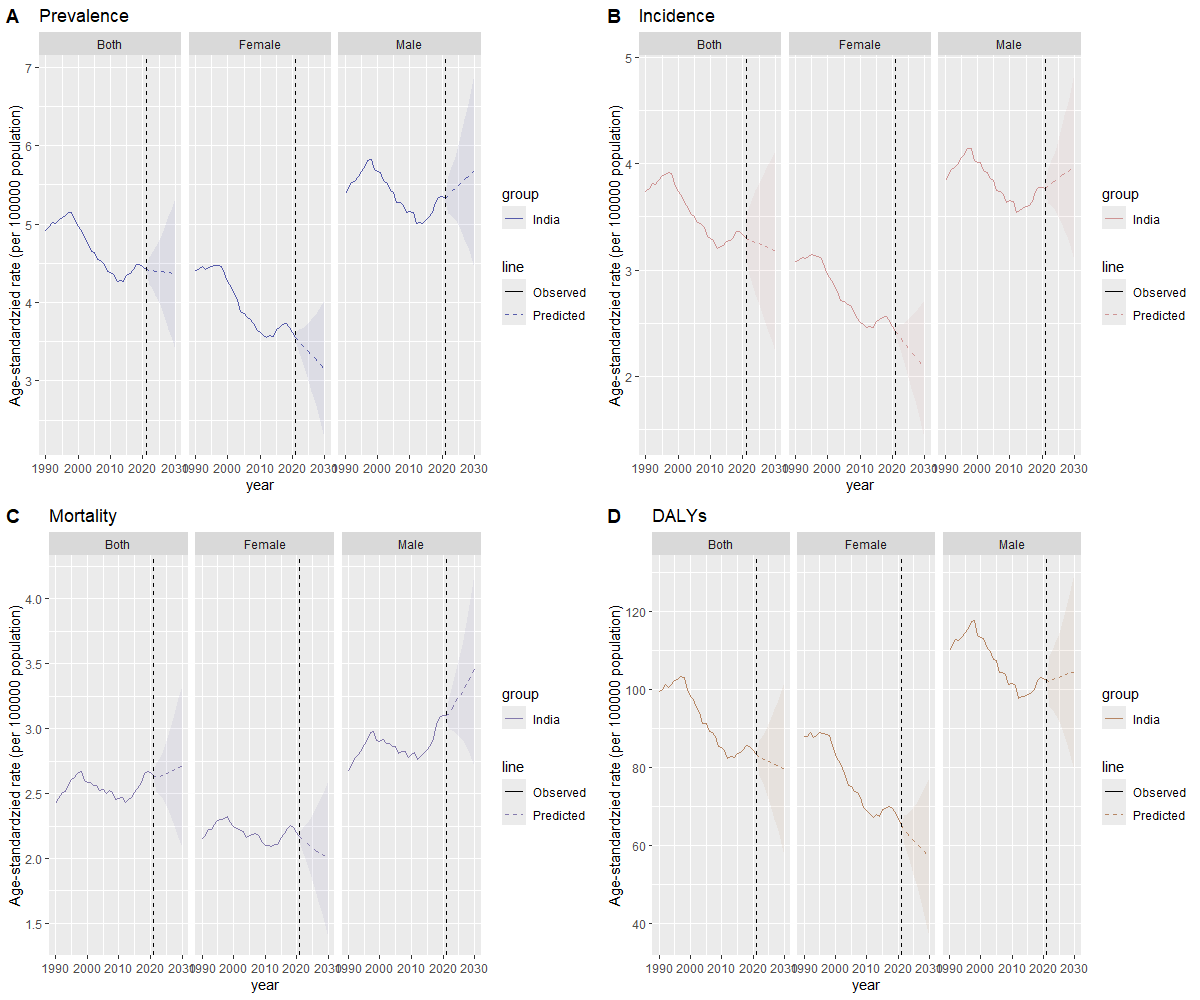
**

**Figure S3 Projection of esophageal cancer ASIR, ASMR, ASMR, and ASDR trends in India from 2021 to 2030.**

**
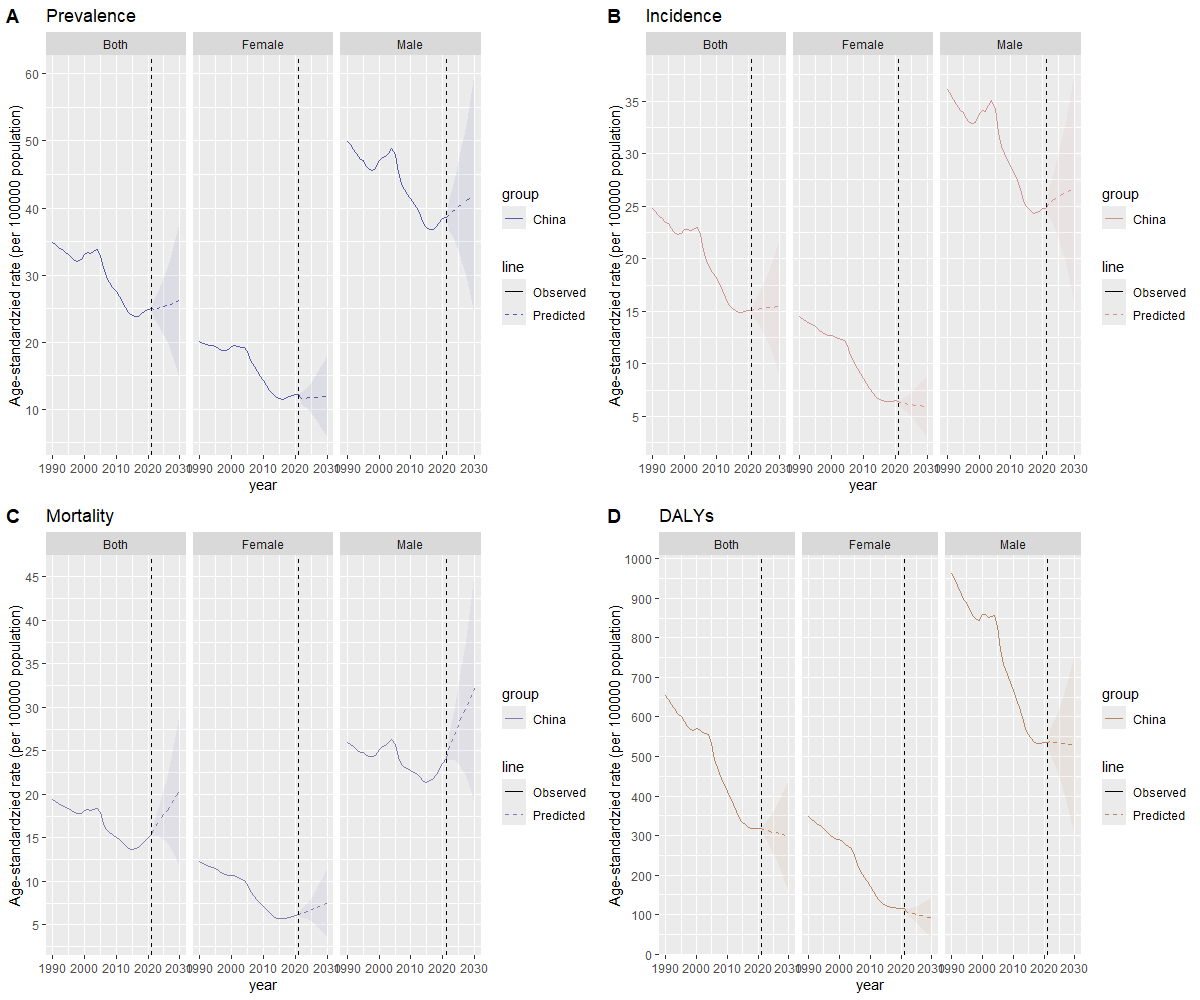
**

**Figure S4 Projection of esophageal cancer ASIR, ASMR, ASMR, and ASDR trends in China from 2021 to 2030.**

**
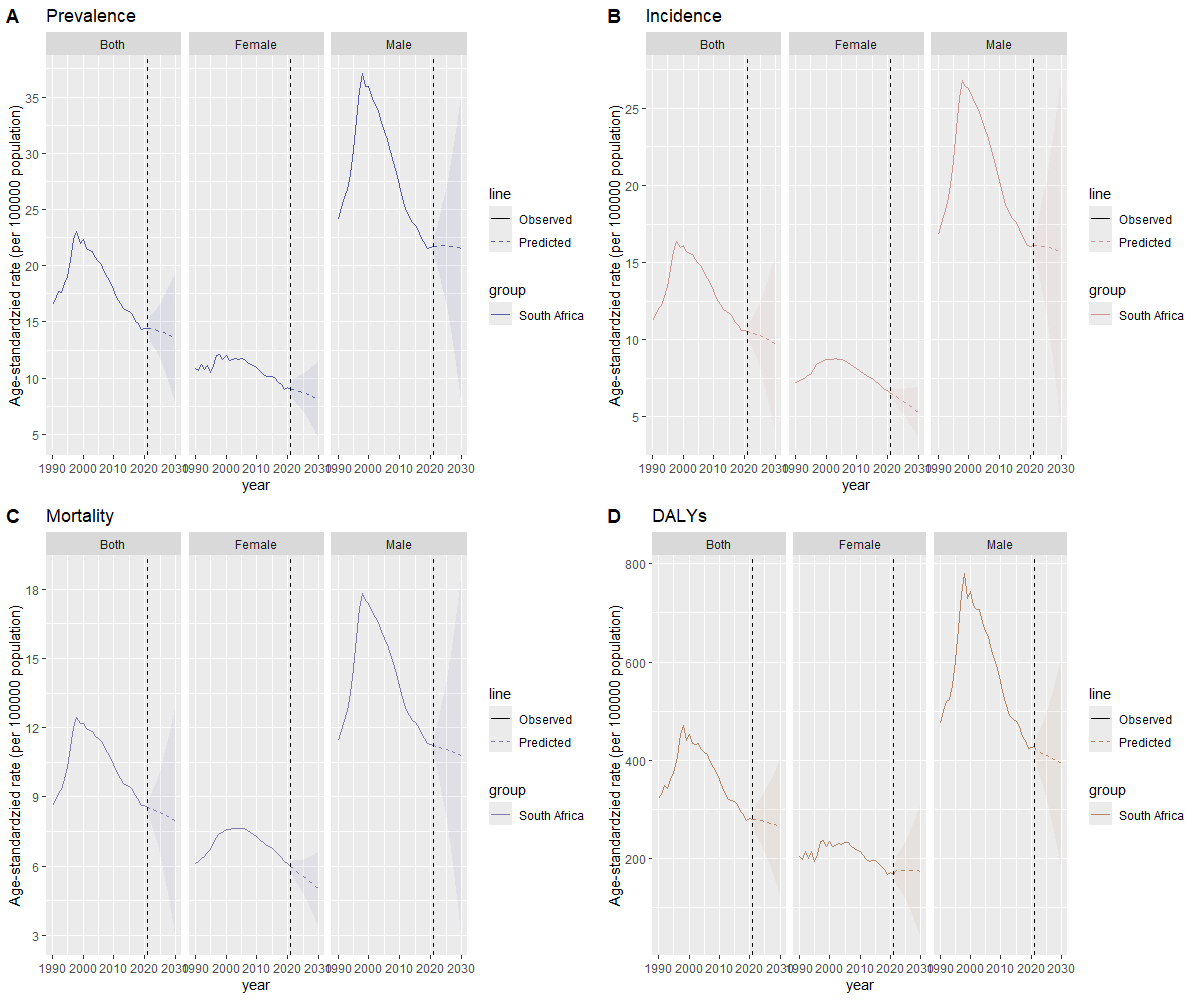
**

**Figure S5 Projection of esophageal cancer ASIR, ASMR, ASMR, and ASDR trends in South Africa from 2021 to 2030.**

**
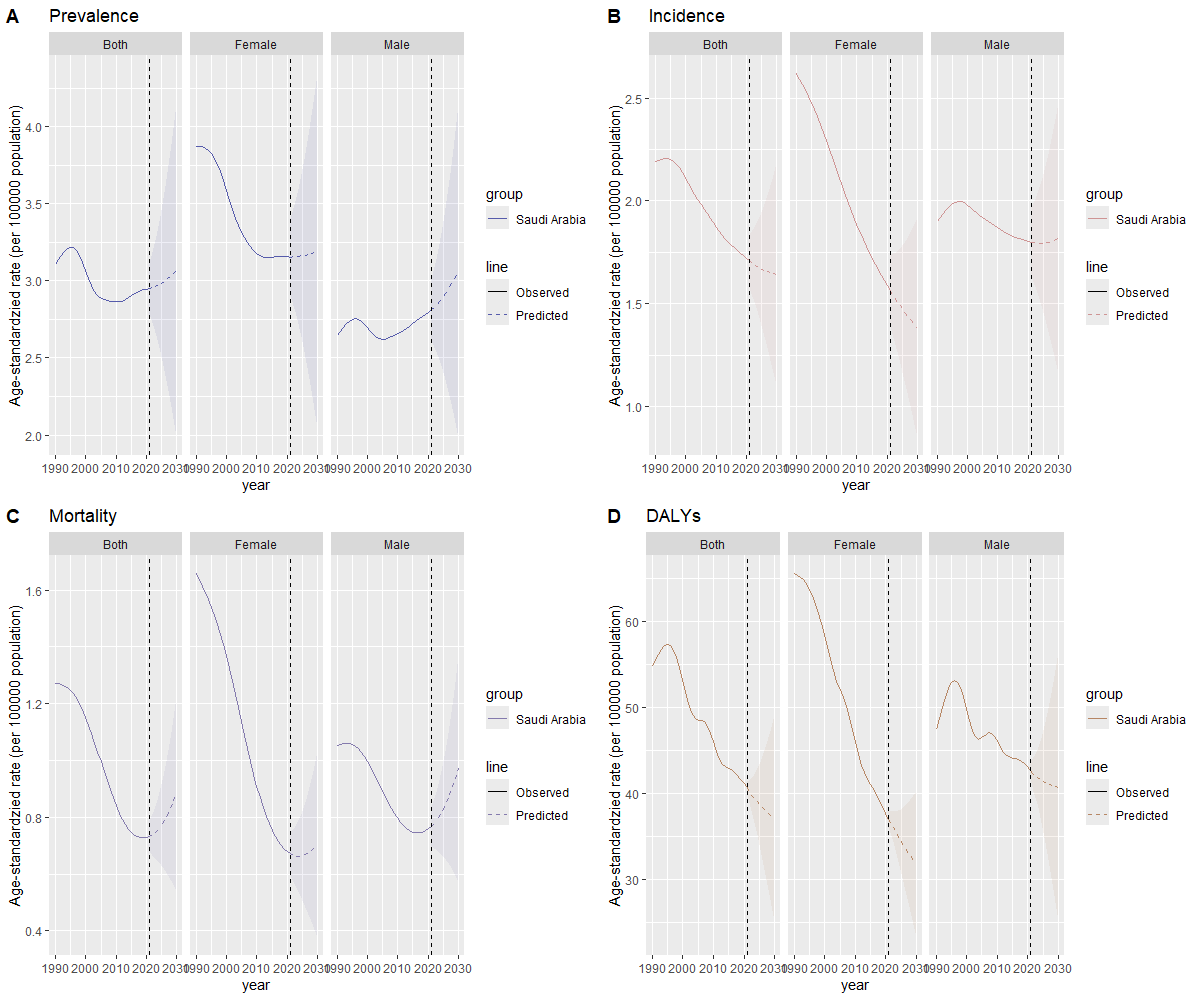
**

**Figure S6 Projection of esophageal cancer ASIR, ASMR, ASMR, and ASDR trends in Saudi Arabia from 2021 to 2030.**

**
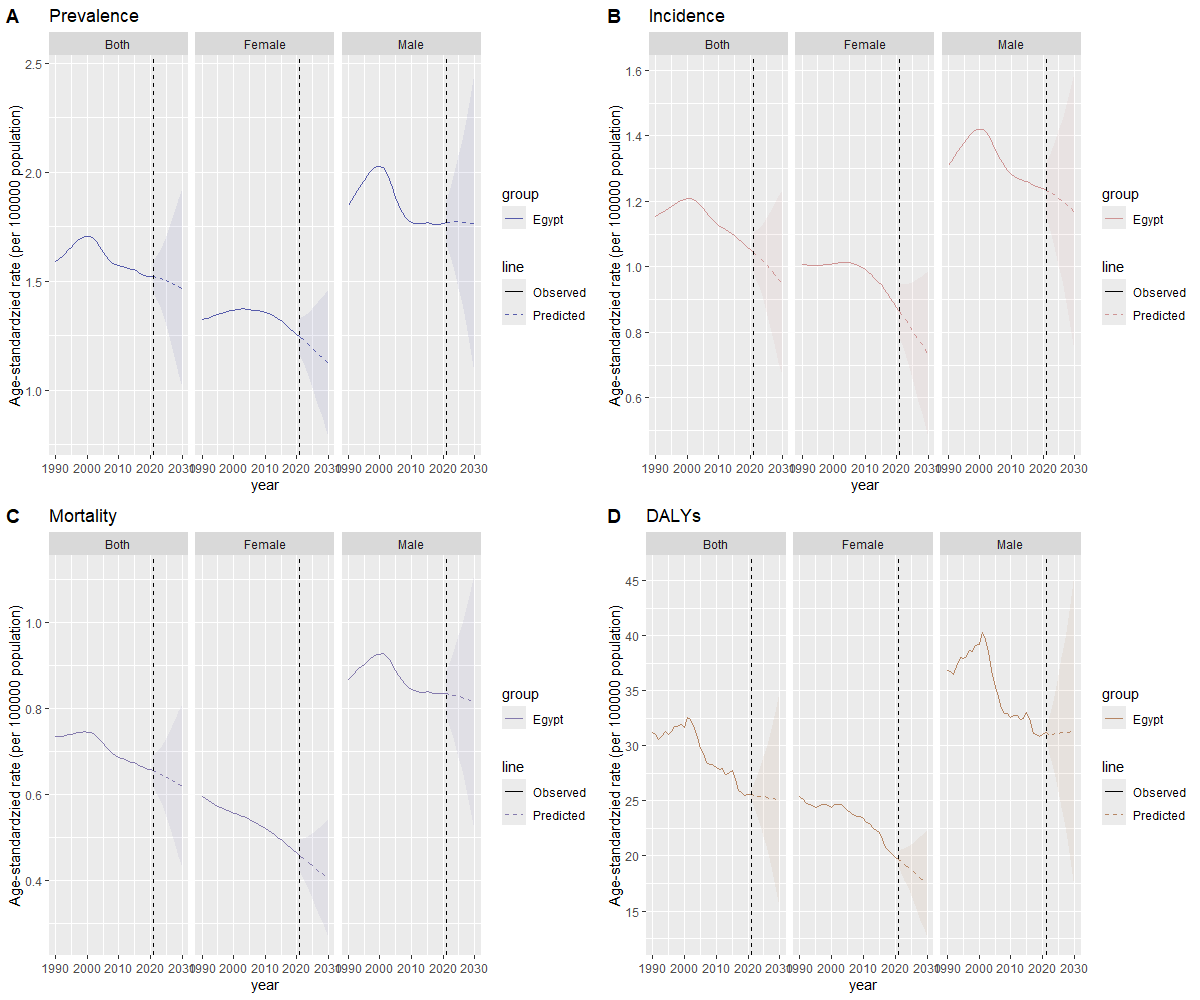
**

**Figure S7 Projection of esophageal cancer ASIR, ASMR, ASMR, and ASDR trends in Egypt from 2021 to 2030.**

**
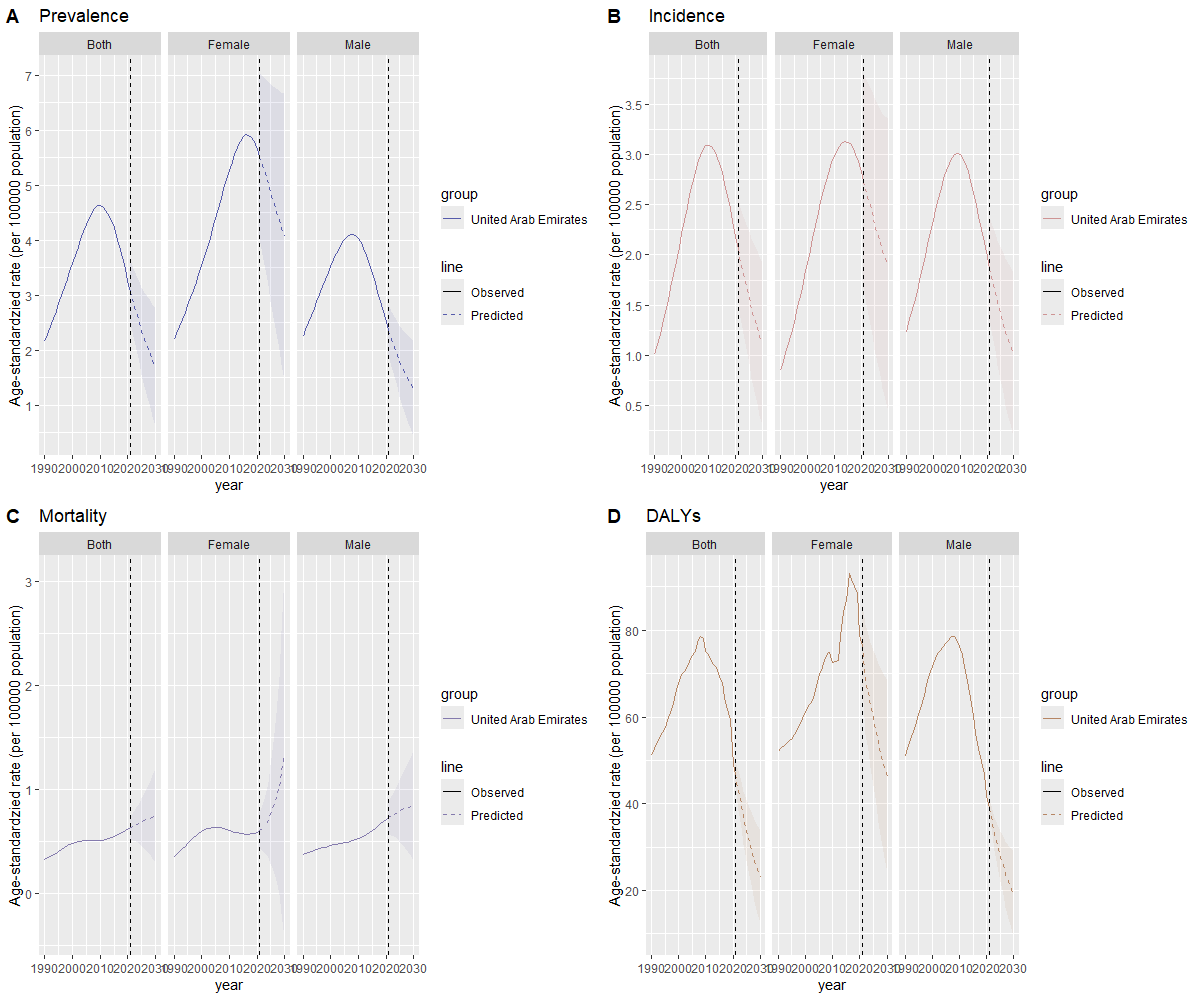
**

**Figure S8 Projection of esophageal cancer ASIR, ASMR, ASMR, and ASDR trends in the UAE from 2021 to 2030.**

**
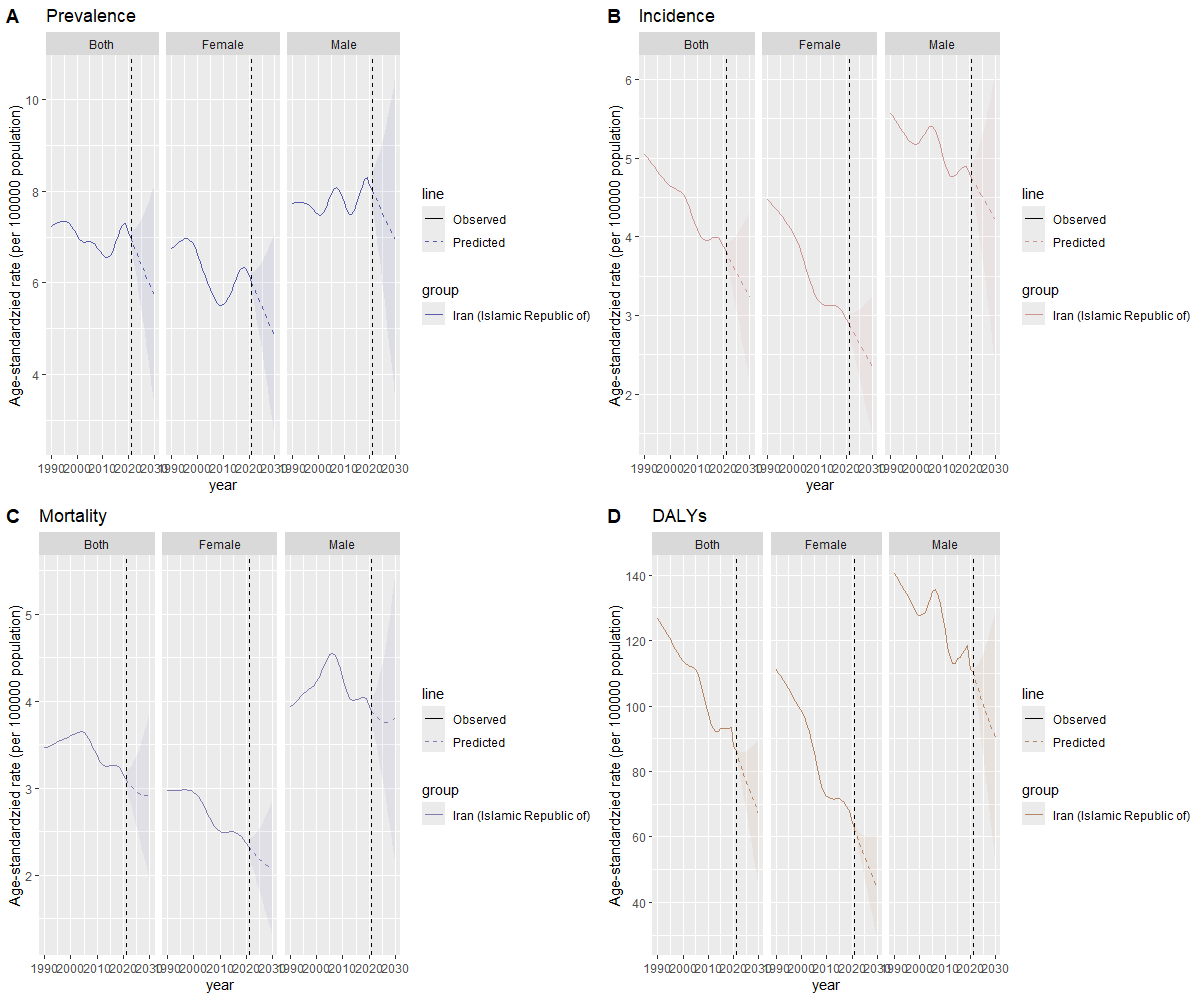
**

**Figure S9 Projection of esophageal cancer ASIR, ASMR, ASMR, and ASDR trends in Iran from 2021 to 2030.**

**
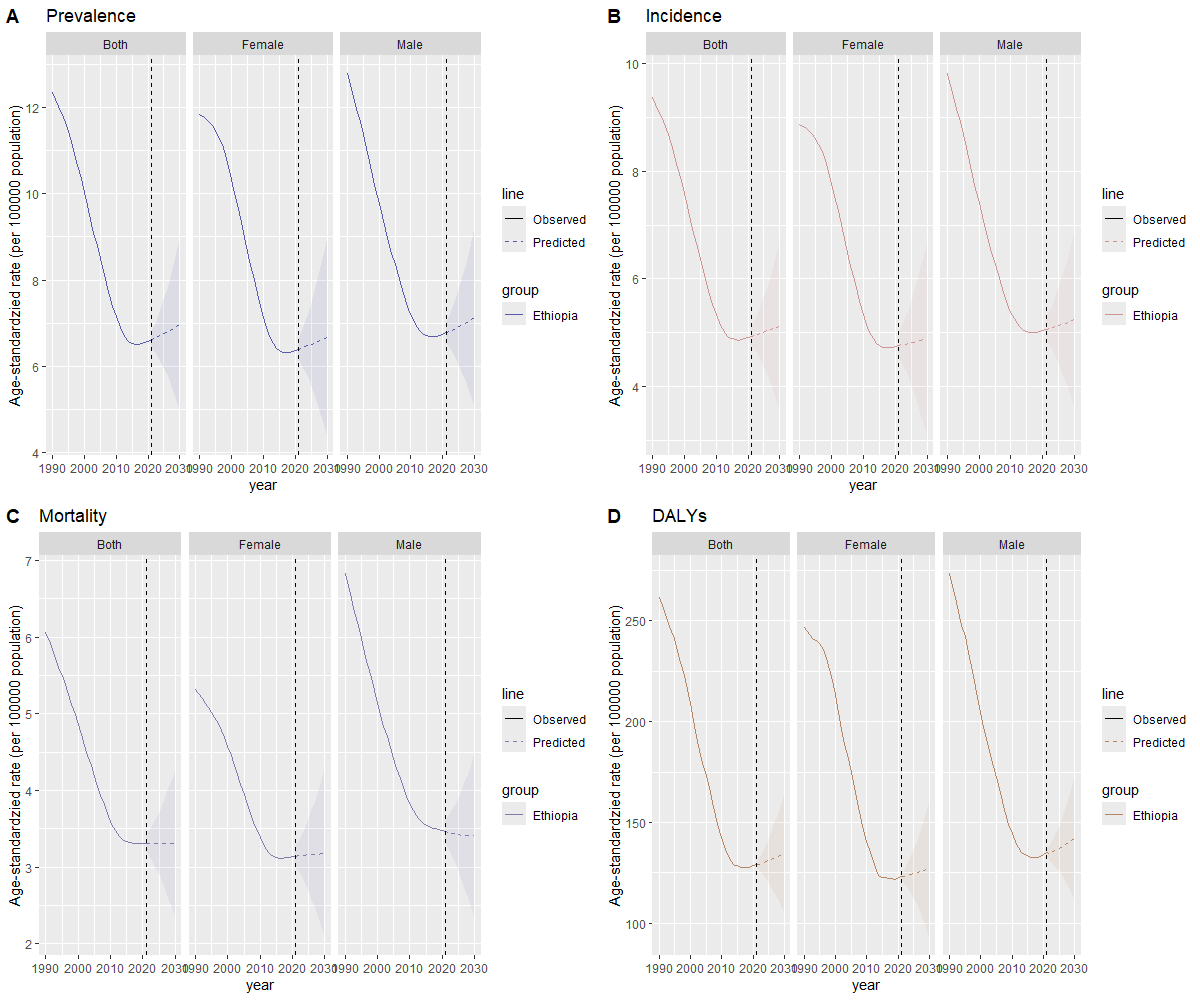
**

**Figure S10** **Projection of esophageal cancer ASIR, ASMR, ASMR, and ASDR trends in Ethiopia from 2021 to 2030.**
